# 非小细胞肺癌脑膜转移的临床病理特征及预后分析

**DOI:** 10.3779/j.issn.1009-3419.2016.08.09

**Published:** 2016-08-20

**Authors:** 敏 朱, 雁宏 任, 艳 刘, 承钧 班, 华 顾, 征 王, 予辉 张

**Affiliations:** 1 100020 北京, 首都医科大学附属北京朝阳医院呼吸与危重症医学科 Beijing Institute of Respiratory Medicine, Department of Respiratory and Critical Care Medicine, Beijing Chao-Yang Hospital, Capital Medical University, Beijing 100020, China; 2 100029 北京, 中日友好医院呼吸与危重症医学科 Department of Pulmonary and Critical Care Medicine, China-Japan Friendship Hospital, Beijing 100029, China; 3 100730 北京, 北京医院病理科 Department of Pathology, Beijing Hospital, Beijing 100730, China

**Keywords:** 肺肿瘤, *EGFR*基因突变, 脑膜转移, Lung neoplasms, *EGFR* mutation, Leptomeningeal metastasis

## Abstract

**背景与目的:**

脑膜转移(leptomeningeal metastasis, LM)是晚期非小细胞肺癌(non-small cell lung cancer, NSCLC)严重并发症之一, 生活质量降低, 预后差。本研究旨在探讨NSCLC-LM患者的临床病理特征及预后。

**方法:**

回顾性分析2015年1月-2016年6月首都医科大学附属北京朝阳医院收治的3例NSCLC-LM患者的临床资料, 并结合文献进行分析。

**结果:**

3例患者均为肺腺癌, 且表皮生长因子受体(epidermal growth factor receptor, EGFR)21外显子L858R突变(mutations, m), 其中男性1例, 女性2例; 年龄59岁-64岁, 平均年龄61.3岁, 主要临床表现及查体:头痛(3/3)、头晕(3/3)、恶心呕吐(3/3)、癫痫(2/3)、复视(1/3)、听力下降(1/3)、脑膜刺激征(3/3)。出现症状到LM确诊时间为1个月-4个月(平均2.3个月)。除了1例肺癌和LM同时诊断, 2例分别在EGFR酪氨酸激酶抑制剂(tyrosine kinase inhibitors, TKIs)和化疗进展后出现LM, 肺癌到LM平均确诊时间为8.5个月。3例患者脑增强磁共振成像(magnetic resonance imaging, MRI)均显示软脑膜线性强化; 3例脑脊液中找到癌细胞, 其中2例行EGFR检测, 均为EGFR 21外显子L858R突变。2例患者接受TKIs治疗, 症状好转, 其中1例联合替莫唑胺, 无进展生存期(progression-free survival, PFS)达4.9个月, 总生存时间(overall survival, OS)为13.9个月。

**结论:**

EGFRm肺腺癌可能易出现LM; NSCLC-LM症状不典型, 易漏诊、误诊; TKIs联合替莫唑胺可能是EGFRm-NSCLC-LM的治疗选择。

## Abstract (en)

**Background and objective:**

Leptomeningeal metastases (LM) is one of the most serious complications of advanced non-small cell lung cancer (NSCLC) due to the lower quality of life and poor prognosis.The aim of this study is to analyze the clinicopathological features and prognosis of patients with LM from NSCLC (NSCLC-LM).

**Methods:**

Clinical data of 3 patients with NSCLC-LM collected from January 2015 to June 2016 were analyzed with a brief review.

**Results:**

All 3 patients had adenocarcinoma histology harboring epidermal growth factor receptor (EGFR) exon 21 point mutations (m).Of the 3 cases, 1 was male, 2 were female.The mean age was 61.3 years (range, 59-64 years).The main clinical manifestations and positive physical examination included headache (3/3), dizziness (3/3), nausea (3/3) and vomiting (3/3), epilepsy (2/3), diplopia (1/3), hearing loss (1/3) and meningeal stimulation sign (3/3).The median time from symptom to diagnosis of LM was 2.3 months (range, 1 to 4 months).Except 1 patient with lung cancer and LM diagnosised at the same time, the other 2 cases received the diagnosis of LM after tyrosine kinase inhibitors (TKIs) therapy or chemotherapy respectively, the median time from diagnosis of NSCLC to LC was 8.5 months.The brain enhanced magnetic resonance imaging (MRI) manifestations of all 3 cases revealed linear meningeal enhancement.Cerebrospinal fluid in 3 cases were positive cytology in whom two cases had EGFR exon 21 L858R mutations, consistenting with the lung tissue.The symptom of the 2 cases improved after TKIs therapy, and temozolomide was used as supplement of 1 case of which the progression-free survival (PFS) and overall survival (OS) was 4.9 months and 13.9 months respectively.

**Conclusion:**

Lung adenocarcinoma with sensitive *EGFR* mutations are likely to appear LM.Lacking of typical symptoms, NSCLC-LM was easily to be missed and misdiagnosed.TKIs therapy combined with temozolomide may be effective therapies for EGFRm-NSCLC-LM patients.

脑膜转移(leptomeningeal metastasis, LM)是恶性肿瘤神经系统转移的一种特殊类型, 发病率低, 预后差。由于临床表现不典型, 早期诊断困难, 易被漏诊、误诊, 严重影响生存期。非小细胞肺癌(non-small cell lung cancer, NSCLC)患者出现LM后平均生存期为14周^[1]^, 因此NSCLC-LM日益受到临床医生的关注。为了解NSCLC出现LM的临床病理特征、表皮生长因子受体(epidermal growth factor receptor, EGFR)酪氨酸激酶抑制剂(tyrosine kinase inhibitors, TKIs)药物治疗及预后的特点, 本文总结3例NSCLC-LM病例报告如下。

## 1 资料与方法

### 1.1 研究对象

收集2015年1月-2016年6月首都医科大学附属北京朝阳医院收治的3例NSCLC-LM患者的临床资料, 男性1例, 女性2例, 年龄59岁-64岁, 平均年龄61.3岁。3例脑脊液(cerebrospinal fluid, CSF)中找到癌细胞, 并且脑增强磁共振成像(magnetic resonance imaging, MRI)表现为LM的典型表现, 符合恶性肿瘤LM的诊断标准。依据2015年NCCN中枢神经系统肿瘤指南(NCCN Clinical Practice Guidelines in Oncology^TM^ Central Nervous System Cancer version 1, 2015), 对于NSCLC患者, 如有新发提示LM的神经系统症状或体征, 发现CSF肿瘤细胞阳性或影像学符合LM典型表现, 可诊断为NSCLC-LM^[2]^。

### 1.2 方法

收集3例患者的临床资料, 分析其临床表现及体征、影像表现、实验室检查、组织及分子病理及治疗经过, 并对患者进行随访。

## 2 结果

### 2.1 临床表现及体征

NSCLC-LM初期症状不典型, 易被漏诊和误诊, 随着疾病进展可表现为颅高压症状。本组患者表现为头痛(3/3)、头晕(3/3)、恶心呕吐(3/3), 出现癫痫(2/3)、复视(1/3)、听力下降(1/3)。体格检查可有脑膜刺激征表现, 本组患者(3/3)均有脑膜刺激征阳性表现(颈强直、Kernig征、Brudzinski征)。3例患者出现症状到LM确诊时间1个月-4个月(平均2.3个月)。除了1例肺癌和LM同时诊断, 另外2例分别在TKIs治疗和化疗进展后出现LM, 肺癌到LM平均确诊时间为8.5个月(表 1)。

**1:** 3例NSCLC-LM患者的临床表现 The clinical manifestations of three NSCLC-LM patients

No.	Gender	Age (year)	Stage	Time from symptoms to diagnosis of LM (months)	Time from diagnosis of lung cancer to LM (months)	Previous EGFR-TKI treatment	Symptoms of intracranial hypertension	Meningeal irritation sign	Epileptic seizure
1	Female	59	cT4N3M1b Ⅳ	4	9	Y	Y	Y	Y
2	Male	61	cT2bN2M1a Ⅳ	1	8	N	Y	Y	Y
3	Female	64	cT4N3M1b Ⅳ	2	At the same time	N	Y	Y	N
NSCLC:non-small cell lung cancer; LM:leptomeningeal metastases; EGFRm:epidermal growth factor receptor mutations; TKI:tyrosine kinase inhibitors; Y:yes; N:no.									

### 2.2 影像表现

3例患者胸部CT均显示右肺肿块, 其中1例患者同时存在胸膜转移(中量胸腔积液), 1例患者存在对侧肺转移。3例患者均行脑增强MRI, 2例患者显示双侧顶叶及额叶软脑膜线性增厚, 脑室扩张, 另1例患者显示右侧小脑幕及小脑蚓部脑膜强化(表 2, 图 1)。

**2:** 3例NSCLC-LM患者的影像学改变 The imaging changes of three NSCLC-LM patients

No.	Position	Pleural effusion	Brain enhanced MRI
1	Right middle and lower lobe	Y	Linear enhancing meninges
2	Right upper and left lower lobe	N	Linear enhancing meninges
3	Right upper lobe	N	The right side of tentorium cerebelli and cerebellar vermis meningeal reinforcement
MRI:magnetic resonance imaging.									

**1:**
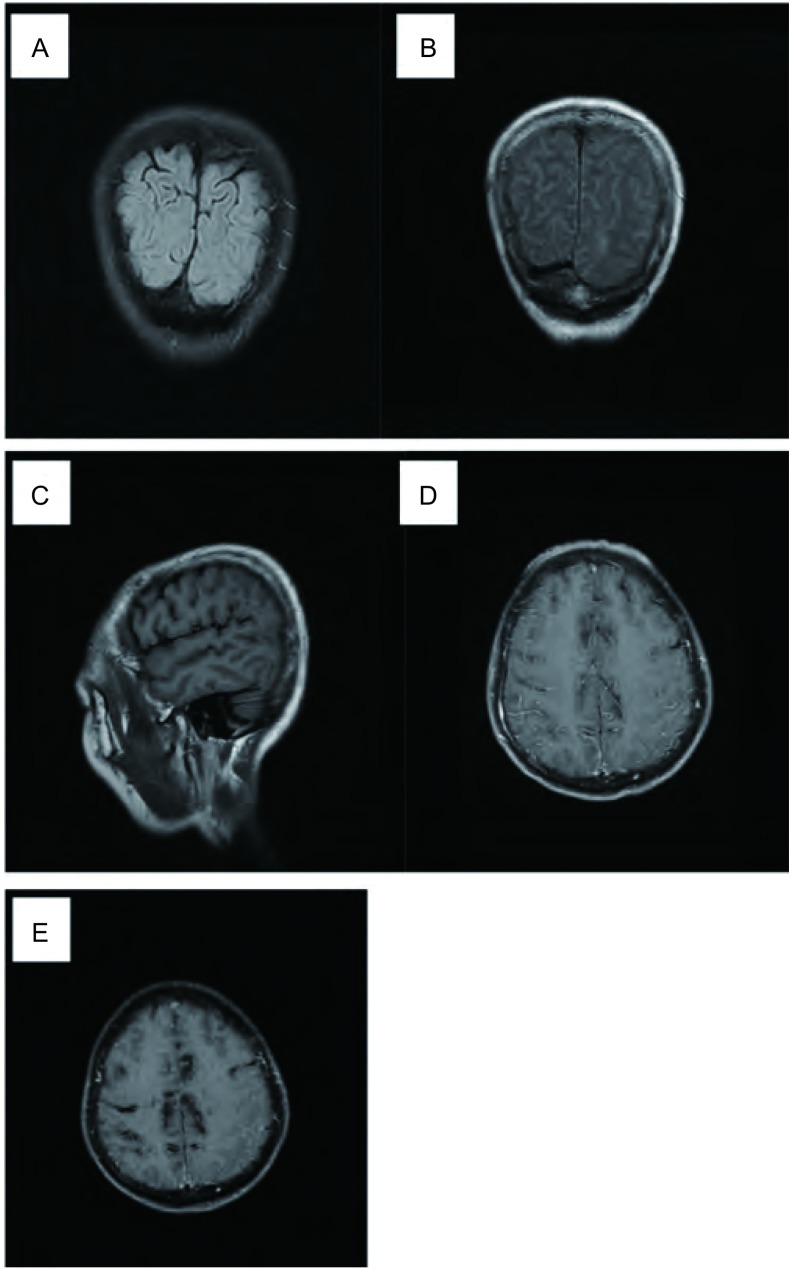
脑MRI改变。A:2015-01-12 T2FLAIR序列未见明确转移灶; B and C:2015-05-17治疗前; MRI:T2FLAIR相冠状位及T1WI矢状位图像与A图像比较, 双侧顶叶脑沟变窄; D:2015-05-17治疗前; MRI:轴位T1WI增强序列显示双侧额顶叶沿脑沟分布的线状明显强化影; 右侧软脑膜增厚并明显强化; E:2015-09-18治疗后; MRI:轴位T1WI增强序列显示, 双侧额顶叶脑沟内线状强化影有所减少。 Patterns of brain MRI.A:No metastases in the T2FLAIR image; B and C:2015-05-17;MRI:before treatment:The coronal T2FLAIR and sagittal T1WI image demonstrated shallow sulcus in bilateral parietal lobe compared with image A; D:2015-05-17;MRI:before treatment:The axial T1WI enhanced image showed linear enhancing meninges along the surface of bilateral frontal and parietal lobe; meninges was thicking and enhancing on the right side; E:2015-09-18 MRI after treatment:The axial T1WI enhanced image showed decreased along the surface of bilateral frontal and parietal lobe.

### 2.3 实验室检查、组织及分子病理

本组患者与腺癌有关的血清肿瘤标记物癌胚抗原(carcinoembryonic antigen, CEA)明显升高。3例患者肺组织的病理均为肺腺癌, 2例行EGFR检测显示21外显子L858R突变, 另外1例由于肺组织少, 未行基因检测, 但发生LM后行CSF的EGFR检测显示21外显子L858R突变。3例患者均接受腰椎穿刺, 发现CSF压力均明显升高, 平均为306.7cmH_2_O, 细胞数和蛋白略增高。3例患者CSF中均找到癌细胞, 2例利用突变扩增阻滞系统(amplification refractory mutation system, ARMS)发现EGFR 21外显子L858R突变, 与肺组织检测结果一致(表 3, 图 2)。

**3:** 3例NSCLC-LM患者的脑脊液和病理学特征 The cerebrospinal fluid and pathological features of three NSCLC-LM patients

No.	Histology	*EGFR* mutation	CEA (ng/mL)	CSF Pressure (cmH_2_O)	Cell count of CSF (/*μ*L)	Protein of CSF (mg/dL)	Glucose of CSF (mmol/L)	Histology of CSF	EGFR mutation of CSF
1	Adenocarcinoma	EGFR 21 L858R	277.2	290	NA	NA	NA	Positive cytology	NA
2	Adenocarcinoma	NA	139.9	330	8.0	46.0	0.34	Positive cytology	EGFR 21 L858R
3	Adenocarcinoma	EGFR 21 L858R	41.59	300	10.0	46.0	3.3	Positive cytology	EGFR 21 L858R
CSF:cerebrospinal fluid; CEA:carcinoembryonic antigen; NA:not available.									

**2:**
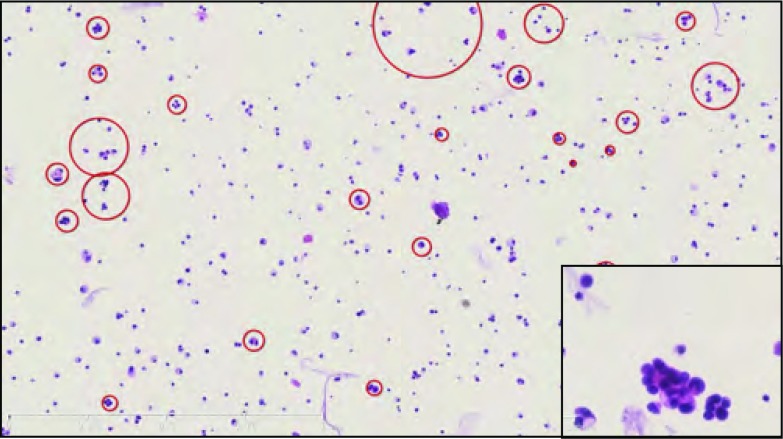
NSCLC-LM患者的脑脊液, 液基薄层细胞学制片HE染色可见大量癌细胞(×50), 右下角为局部放大图(×400)。 Cerebrospinal fluid from NSCLC-LM patient.Almost all of the cells in the thin-preparation cytologic test slide were cancerous cells (the red circles) (HE staining, ×50), and magnification of figure was inthebottom right corner(HE staining, ×400).

### 2.4 治疗和转归

3例患者确诊时, 一般情况差, 均拒绝行鞘内注射化疗及全脑放疗。1例在服用吉非替尼9个月后确诊LM, 将吉非替尼改为厄洛替尼150 mg *qd*, 并联合替莫唑胺(temozolomide)200 mg *qd* *po* d1-d5, 28天为一疗程, 治疗4个月后头痛症状较前改善, 复查脑增强MRI显示局部脑膜好转, 但终因大量胸腔积液呼吸衰竭死亡, 无进展生存期(progression-free survival, PFS)为4.9个月, 总生存时间(overall survival, OS)达13.9个月。1例患者先后接受紫杉醇脂质体联合顺铂治疗4周期、多西他赛120 mg单药化疗方案2周期, 出现颅高压症状, CSF中EGFR21外显子L858R突变, 患者拒绝接受TKIs药物, 1个月后死亡。1例患者同时发现肺组织和CSF的EGFR21外显子L858R突变, 给予口服吉非替尼250 mg *qd*, 1个月后头痛、头晕好转, 目前随访中(表 4)。

**4:** 3例NSCLC-LM患者的治疗和预后 The treatment and prognosis of three NSCLC-LM patients

No.	PS	EGFR-TKIs treatment for LM	PFS (month)	OS (month)	Prognosis
1	3	Erlotinib+temozolomide	4.9	13.9	Death
2	4	N	NA	8	Death
3	4	Gefitinib	1	NA	Follow-up
PS:performance status; PFS:progression-free survival; OS:overall survival.					

## 3 讨论

恶性肿瘤LM又称为脑膜癌病(meningeal carcinomatosis, MC), 指全身各部位恶性肿瘤细胞在脑膜上弥漫而广泛的播散及种植, 伴或不伴有脑实质和脊髓的转移性肿瘤, 是恶性肿瘤神经系统转移的一种特殊类型。目前认为NSCLC-LM的发病率在10%-26%^[3]^。

LM起病隐匿, 临床表现缺乏特异性。最常见的症状是颅高压和脑膜刺激症状, 如头痛、恶心和呕吐, 其他如癫痫发作、无力、感觉失常、复视、听力下降等^[1]^。本文中3例患者的首发症状均为头痛、恶心、呕吐, 部分患者出现癫痫发作, 并表现脑膜刺激征阳性, 与文献中一致。通常确诊NSCLC到LM的中位时间是10.7个月, 提示LM往往是肺癌晚期表现, 并且60.9%的患者之前接受过TKIs^[4]^。本文除了1例NSCLC和LM同时诊断, 另外2例患者肺癌确诊距LM诊断的平均时间为8.5个月, 并且分别出现在TKIs治疗前后, 与文献中的相似。另外本文中3例患者从出现症状到LM确诊时间平均2.3个月, 在此期间常常认为头痛等症状为非特异性表现, 延误诊断。

由于NSCLC-LM患者一般情况差, 无创的脑增强MRI扫描在LM的诊断中显得更加重要。国外研究报道, 经CSF细胞学病理证实LM, 2/3患者的脑增强MRI有阳性发现^[5]^, 甚至有研究^[1]^发现94%的脑增强MRI可见相应改变, 表现为蛛网膜点线样强化、脑沟回内转移结节(皮质转移)、马尾神经种植结节等。本文的3例患者脑增强MRI均显示沿脑膜分布的线状强化影, 符合LM的影像特点。

病理诊断是LM诊断的重要部分。确诊的NSCLC-LM中腺癌是最常见的病理类型, 大约占84%-97%^[2]^, 并且*EGFR*突变率在74.3%, 明显高于亚裔人群平均的突变率^[4]^, 提示突变人群更可能发生LM。本文中3例患者均为*EGFR*敏感突变肺腺癌, 符合文献报道, 其主要原因是药物难以通过血脑屏障达到有效治疗浓度。以吉非替尼为例, CSF与血浆浓度比为1.3%±0.7%^[6]^, 意味着很少量的TKIs通过血脑屏障。研究^[7, 8]^发现EGFRm-NSCLC-LM患者的CSF基因突变与肺组织一致, 并未检测到常见的耐药突变T790M, 其结果支持血脑屏障是发生LM的主要原因。仅有少数病例发现CSF中T790M^[7, 9]^的突变。因此实时动态观察CSF的基因突变状态才是找到LM真正原因的方法。

LM诊断的金标准仍是CSF病理学检查。利用新技术提高CSF的诊断率, 如免疫荧光染色-染色体荧光原位杂交(TM-iFISH)技术检测CSF循环肿瘤细胞^[10]^, 直接DNA测序法和实时PCR法检测*EGFR*突变情况^[8]^。本文3例患者均为首次腰穿发现癌细胞, 2例采用ARMS方法对CSF进行EGFR检测发现21外显子L858R突变, 从而指导治疗。

LM如不进行治疗, 生存期仅4周-6周^[11]^, 接受治疗总的生存期可延长到3个月-6个月。鞘内注射化疗和全脑放疗仍是肺癌LM的传统治疗方案, 但疗效欠佳。多个病例报道提示TKIs药物治疗EGFRm-NSCLC-LM有效^[12, 13]^, 中位OS可达19.2个月^[14]^; 并且无论在诊断LM前或诊断后接受TKIs治疗的患者较未接受者, 均能延长中位OS(10.9个月*vs* 2.3个月, *P* < 0.001)^[4]^。

不同的TKIs疗效有差异。由于厄洛替尼的血脑屏障通过率高, 在CSF中可达到有效抗肿瘤浓度^[15-17]^, 因此厄洛替尼组CSF细胞学转阴率高(64.3% *vs* 9.1%, *P*=0.012), OS较吉非替尼组延长(9.5个月 *vs* 4.4个月)^[18]^。另外具有更好的血脑屏障穿透力的奥斯替尼(NCT02228369)和AZD3759(NCT02228369), 目前已显示对耐药的EGFRm-NSCLC-LM具有抗肿瘤活性效果, 也可作为选择。本文的1例患者在应用吉非替尼中出现LM, 考虑到血脑屏障的因素, 更换为厄洛替尼, 并联合替莫唑胺治疗, 因此OS明显好于其他患者。替莫唑胺常用于治疗脑胶质细胞瘤, 对血脑屏障具有较好的通透性, CSF中的浓度-时间曲线下面积是血浆中的20%。替莫唑胺治疗LM的Ⅱ期、非随机、多中心、前瞻性研究, 其中肺癌占37%, 中位生存期为43天^[19]^。从初步的研究结果看, 肺癌LM应用替莫唑胺耐受性好, 未影响生活质量。替莫唑胺与TKIs小分子药物合用治疗EGFRm-NSCLC-LM并不多见, 从本文中的数据看, 不排除替莫唑胺与TKIs药物协同穿透血脑屏障, 发挥抗肿瘤活性, 但其药理机制及临床疗效需要进一步的探索研究。

关于NSCLC-LM预后, 研究显示体力活动状态(performance status, PS)评分差、CSF中蛋白及白细胞升高是预后差的预测因素, 而鞘内注射化疗、EGRF-TKIs和全脑放疗是预后良好的预测因素^[1]^。对于EGFRm-NSCLC-LM, 研究显示确诊时PS评分在0-1分生存期更可能超过6个月, 并且较PS≥2分的患者生存期明显延长^[7]^。本文中的1例患者PS评分优于其他患者, 总生存也好于其他患者, 提示早期诊断, 在PS评分良好时接受治疗者预后好。

本研究的不足之处在于是回顾性研究, 并且病例数少, 因此需要进一步积累样本数, 期待得到更有意义的数据。关于替莫唑胺在脑膜转移中的疗效仍需要前瞻性、多中心、大样本研究证实。

总之, 肺癌LM临床症状不典型, 易漏诊或误诊。当EGFRm-NSCLC接受TKIs治疗前或治疗后出现头痛、恶心、呕吐等颅高压及脑膜刺激征等表现, 并且脑增强MRI未见脑实质转移或仅有微小转移, 这种临床症状和脑部受累不相匹配时, 高度提示LM可能, 可以再次复阅影像, 必要时进行MRI脑膜强化的影像学检查, 发挥多学科合作, 尽早完善CSF病理检查, 利用TM-iFISH技术检测循环肿瘤细胞或ARMS法检测*EGFR*基因均可提高其诊断率, 并为后续治疗做准备, TKIs药物联合替莫唑胺可能是EGFRm-NSCLC-LM未来治疗的选择。
